# *Puccinia triticina* pathotypes THTT and THTS display complex transcript profiles on wheat cultivar Thatcher

**DOI:** 10.1186/s12863-020-00851-5

**Published:** 2020-04-28

**Authors:** Jie Wei, Liping Cui, Na Zhang, Dongdong Du, Qingfang Meng, Hongfei Yan, Daqun Liu, Wenxiang Yang

**Affiliations:** 1 Department of Plant Pathology, Hebei Agricultural University/Technological Innovation Center for Biological Control of Plant Diseases and Insect Pests of Hebei Province/National Engineering Research Center for Agriculture in Northern Mountainous Areas, Baoding, 071001 China; 2 Graduate School of Chinese Academy of Agricultural Sciences, Beijing, 100081 China

**Keywords:** Wheat leaf rust, RNA-seq analysis, Effector proteins, Differential expression analysis, qRT-PCR

## Abstract

**Background:**

Wheat leaf rust is an important disease worldwide. Understanding the pathogenic molecular mechanism of *Puccinia triticina* Eriks*.* (*Pt*) and the inconstant toxic region is critical for managing the disease. The present study aimed to analyze the pathogenic divergence between *Pt* isolates.

**Results:**

Total RNA was extracted from the wheat cultivar Thatcher infected by two *Pt* isolates, Tc361_1 (THTT) and Tc284_2 (THTS), at 144 h post inoculation (hpi). The mRNA was then sequenced, and a total of 2784 differentially expressed genes (DEGs) were detected. Forty-five genes were specifically expressed in THTT; these genes included transcription initiation factors and genes with transmembrane transporter activity and other genes. Twenty-six genes were specifically expressed in THTS, including genes with GTPase activity, ABC transporters and other genes. Fifty-four differentially expressed candidate effectors were screened from the two isolates. Two candidate effectors were chosen and validated on tobacco, and the results showed that they could inhibit necrosis induced by BAX. qRT-PCR of 12 significant DEGs was carried out to validate that the results are similar to those of RNA-seq at 144 hpi, to show the expression levels of these DEGs in the early stage and to elucidate the differences in expression between the two *Pt* pathotypes.

**Conclusion:**

The results obtained in this study showed that although the two pathotypes of THTT and THTS contribute similar virulence to wheat, there are a large number of genes participate in the interaction with the susceptible wheat cultivar Thatcher, and revealed the pathogenicity of rust is very complicated.

## Background

Wheat leaf rust caused by *Puccinia triticina* Eriks*.* (*Pt*) develops on approximately 15 million hectares of wheat annually in China, causing a yield loss of approximately three million tons [1]. Significant selection pressure on the fungal pathogen populations enables them to overcome the resistance of wheat cultivars grown in monoculture. Studying the pathogenic mechanism of wheat leaf rust has become an urgent matter to prevent pathogen infection.

Like other phytopathogenic organisms, the leaf rust fungus absorbs nutrients from wheat and exchanges information through haustoria [2], which are nutrient organs that induce structural changes, such as cytoskeletal reorganization, nuclear transplantation and chromatin condensation, in host cells [3]. Haustoria also affect the metabolism of host cells [4, 5]. In addition, haustoria secrete effector molecules into the extrahaustorial matrix that are then transported to the host cells to alter plant cellular defense, architecture and metabolism, ultimately leading to compatible plant-pathogen interactions [6, 7].

The rust genome has been estimated to be 100–135 Mbp in size [8, 9]. Since the genome is so large and 43% of the sequences are repeats, assembly and analysis are challenging. Sixty-nine genes induced as the fungus developed on plants four days after inoculation were identified by using suppression subtractive hybridization (SSH) [10]. The proteomes associated with susceptible wheat/*Pt* interactions have been investigated using two-dimensional polyacrylamide gel electrophoresis (2D-PAGE) at nine days post inoculation [11]. A database of expressed sequence tags (ESTs) representing several life cycle stages of *Pt* have been developed [12]. Furthermore, PtMAPK was found involved in pathogen development, as are its orthologues in *Ustilago maydis* [13]. Ten potential avirulence candidates were found among 432 ESTs derived from haustoria and infected plants in a study identifying the wheat leaf rust genes expressed during the early stages of infection [14].

RNA-seq is useful for revealing information regarding the transcriptome of *Pt*. *Melampsora larici-populina* (*Mlp*) 98AG31 and *Puccinia graminis* f. sp. *tritici* (*Pgt*) SCCL were sequenced, and 16,399 and 17,773 secreted proteins were found, respectively [15]. Five candidate effector proteins were identified with polymorphisms among 2999 secreted proteins by sequencing the different virulence of stripe rust races PST-87/7 and PST-08/21 from the UK [16]. Six different *Pt* races interacted with wheat six days after inoculation were sequenced using the Illumina platform [17]. Five hundred and thirty-two candidate secreted proteins were predicted, of which 456 were present in the tests for all the races, and twelve candidate avirulent genes were predicted. A comparative genomic approach was integrated with an association analysis to identify candidate effector genes corresponding to *Lr20* in phenotype-paired *Pt* isolates from Australia [18]. In that study, twenty *Pt* isolates comprising 10 phenotype-matched pairs with contrasting *Lr20* pathogenicity were analyzed using whole-genome sequencing. The study was the first to integrate phenotype-genotype associations with effector prediction in *Pt* genomes, and the approach used may circumvent the technical difficulties associated with working with obligate *Pt* and accelerate the identification of avirulence genes. *AvrSr35* and *AvrSr50* were cloned, and mutation, RNA-seq and genome sequencing have been conducted on isolates of *Pgt* [19, 20]. The discovery of *AvrSr35* and *AvrSr50* not only provided new tools for the identification of *Pgt* avirulence genes and the characterization of the molecular determinants of immunity in wheat but also indicated the complexity of the pathogenic mechanism of *Pgt*. Relevant studies investigating candidate effector proteins have been performed internationally. Although there is great progress in rust fungus research, understanding of the pathogenic mechanisms is limited. Elucidation of the differential gene expression among different *Pt* races on the same susceptible plant will help reveal differences in pathogenicity among Chinese *Pt* isolates. Thus, to gain more insight into the molecular basis of *Pt*/wheat and to identify genes involved in the pathogenicity and virulence, RNA-seq was conducted at 144 hpi to analyze the DEGs between two *Pt* pathotypes, THTT and THTS.

## Methods

### RNA preparation and establishment of the transcriptome library

Approximately 20 seeds of the susceptible wheat cultivar Zhengzhou 5389 (provided by our laboratory) were planted in a pot with equal diameter and height (10 cm) in a greenhouse. Seven-day-old seedlings were artificially inoculated separately with the *Pt* pathotypes THTT and THTS (provided by our laboratory), which are epidemic strains that differ only in the final “T” or “S” (Table 1). The inoculated plants were placed in a moist chamber overnight at 18–20 °C in the dark and then transferred to greenhouse chambers and cultured at 20 ± 2 °C for 12 h under light. The *Pt* pathotypes were purified, propagated on Zhengzhou 5389 plants and then inoculated onto the susceptible Thatcher line. We sampled the same positions of the inoculated Thatcher leaf tissue at 144 hpi for extraction of total RNA of *Pt*. The RNA was isolated using an RNA isolation kit (RNeasy Plant Mini Kit, Tiangen, Beijing) according to the manufacturer’s instructions. An Illumina HiSeq 2000 sequencing platform was used to sequence the total RNA (sample IDs for THTT and THTS: Tc361_1 and Tc284_2, respectively) after testing the quality of the extracted RNA (conducted by the BGI Company).

**Table 1:** Virulence formulas of THTS and THTT

pathotype	Virulence formula (low virulence/high virulence)
THTS	(*Lr9 Lr18 Lr19 Lr24 Lr38 Lr39 Lr42 Lr47*)/(*Lr1 Lr2a Lr2b Lr2c Lr3 Lr3bg Lr3ka Lr10 Lr11 Lr14a Lr14b Lr15 Lr16 Lr17 Lr20 Lr21 Lr23 Lr25 Lr26 Lr28 Lr29 Lr30 Lr32 Lr33 Lr36 Lr37 Lr44 Lr45 Lr46*)
THTT	(*Lr9 Lr19 Lr24 Lr36 Lr38 Lr39 Lr42 Lr44 Lr47*)/(*Lr1 Lr2a Lr2b Lr2c Lr3 Lr3bg Lr3ka Lr10 Lr11 Lr14a Lr14b Lr15 Lr16 Lr17 Lr18 Lr21 Lr23 Lr25 Lr26 Lr28 Lr29 Lr30 Lr32 Lr33 Lr37 Lr45 Lr46 Lr50*)

### Data analysis

The DEGs according to the sequencing data were screened using the reads per kilobase per million reads (RPKM) algorithm [21] and a threshold value. We considered genes with false discovery rate (FDR) less than or equal to 0.001 and differences of less than 2-fold to be DEGs, and we annotated the functions and signaling pathways of these genes via Gene Ontology (GO) and Kyoto Encyclopedia of Genes and Genomes (KEGG) pathway analysis. The data were prepared for cluster analysis, and the secreted proteins were predicted by SignalP 4.1, TMHMM 2.0 and EffectorP 2.0 [22]. Additionally, functional annotation was performed with the Nonredundant (Nr) database. Known motifs, including RxLR in oomycetes, [Y/F/W] xC in powdery mildew and G [I/F/Y][A/L/S/T] R in flax rust, were identified with Perl software. The conserved domains were identified using Pfam software.

### qRT-PCR analysis

The expression of the DEGs and the primers used for qRT-PCR are presented in Table S1. Leaves were infected with one of the two different pathotypes of *Pt*-THTT and THTS and sampled at 0, 6, 12, 18, 24, 36, 48, 72, 96, 144, 216 hpi and 288 hpi. The fluorescence quantitative analysis was performed using a Roche LightCycler 96 real-time PCR instrument. The experiments were conducted with three biological repeats, and actin-β was used as an internal reference gene. The PCR conditions were as follows: 5 min at 94 °C; 40 cycles of 10 s at 94 °C, 15 s at 60 °C and 20 s at 72 °C; one cycle of 10 s at 95 °C, 60 s at 65 °C and 1 s at 97 °C; and a final step of 30 s at 37 °C. The relative expression levels of the target genes at the different time points were calculated using the 2^-ΔCT^ method.

### Agrobacterium-mediated transient transformation of *Nicotiana benthamiana*

Two candidate effectors were chosen and tested for their ability to inhibit cell death induced by BAX. The recombinant plasmids PGR107::*Unigene17565* and PGR107::*Unigene23118*, which were constructed using primers extended with a *Cla*I and an *Xma*I site, respectively (Table S2), were transferred into GV3101 *Agrobacterium*-competent cells, and the positive clones were picked and shaken at 28 °C. The clones and the cell death inducer (BAX) were diluted to an optical density at OD_600_ value of 0.3. The 2nd to 4th leaves of 6- to 8-week-old *N. benthamiana* plants were infiltrated by the effectors and BAX at the same time. The leaves were photographed 7 days after inoculation.

## Results

### Plant response to infection

The only differences between strain THTS and strain THTT were the “S” and “T” in the corresponding pathotypes, which meant that THTS was avirulent to *Lr18* in the virulence formula, while THTT was virulent to *Lr18*. In addition, THTT was avirulent to *Lr36* and *Lr44* in the virulence formula, while THTS was virulent to them (Table 1).

### DEGs based on RNA-seq

**Fig. 1:**
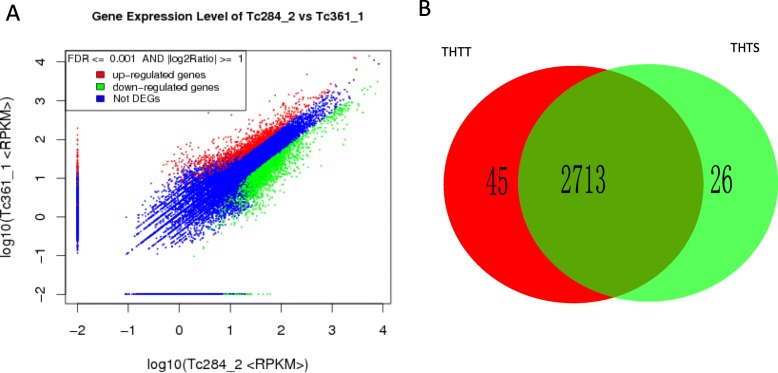
The overview of differential expression of genes in THTT and THTS. **a**. The expression level of genes in THTT and THTS. **b**. The number of specially expressed genes in THTT and THTS

The total numbers of reads obtained from the databases for THTS and THTT were 7,206,771 and 7,040,346, respectively. The gene expression levels were compared between THTT as the treatment group and THTS as the control group. A total of 21,172 genes with different expression levels, including 1367 genes that were specifically expressed in THTT and 1222 genes that were specifically expressed in THTS, were screened. In addition, 2784 significant DEGs (FDR ≤0.001 and a difference ≥ 2-fold), of which 1708 were upregulated and 1076 were downregulated (Fig. 1a), were obtained. In total, forty-five and twenty-six DEGs were expressed specifically in THTT and THTS, respectively. (Fig. 1b). Few of them were annotated including zinc metalloprotease, phospholipase, RNA polymerase, cell wall protein, actin cytoskeleton-regulatory complex protein and glycoprotein glucosyltransferase among the upregulated genes (Table S3), and phosphate acyltransferase, dihydroneopterin aldolase and adenosine triphosphatase (ATPase) among the downregulated genes (Table S4).

In THTT vs THTS, a total of 2784 DEGs were enriched for terms in the three GO functional categories: biological processes, cellular components and molecular functions (Table 2). A total of 875 genes were annotated to the biological process category, 539 genes were annotated to the cellular component category, and 971 genes were annotated to the molecular function category.

**Fig. 2:**
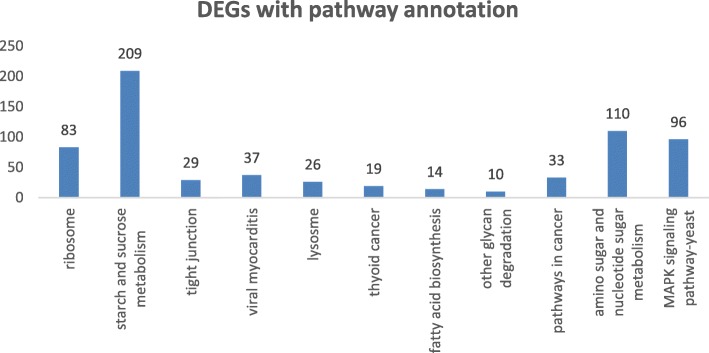
The KEGG pathwayenrichment of DEGs

**Table 2:** Results of GO analysis of 2784 DEGs between THTS and THTT

Ontology category	Class	No. of DEGs	Ontology category	Class	No. of DEGs
Biological process	Biological adhesion	1	Cellular component	Cell	425
	Biological regulation	126		Cell part	425
	Cellular component organizationor biogenesis	69		Macromolecular complex	216
				Membrane	186
	Cellular process	652		Membrane part	142
	Establishment of localization	169		Membrane-enclosed lumen	15
	Developmental process	9		Nucleoid	2
	Growth	1		Organelle	271
	Localization	173		Organelle part	119
	Metabolic process	656	Molecular function	Antioxidant activity	5
	Multiorganism process	1		Binding	579
	Multicellular organismal process	3		Catalytic activity	615
	Negative regulation of biological process	9		Electron carrier activity	4
				Enzyme regulator activity	12
	Positive regulation of biological process	4		Guanyl-nucleotide exchange factor activity	19
	Regulation of biological process	118		Molecular transducer activity	13
	Single-organism process	426		Nucleic acid binding transcription factor activity	17
	Reproductive process	2		Protein binding transcription factor activity	1
	Response to stimulus	94		Receptor activity	6
	Signaling	61		Structural molecule activity	88
	Reproduction	4		Transporter activity	75

In the KEGG pathway analysis, a Q value less than or equal to 0.05 was taken to indicate that a DEG was significantly enriched. In total, 1562 genes among 2784 DEGs were annotated to 153 KEGG pathways. The pathways with the highest enrichment confidence, including ribosome, starch and sucrose metabolism, tight junction, viral myocarditis, lysosome, thyroid cancer, fatty acid biosynthesis, other glycan degradation, pathways in cancer, amino sugar and nucleotide sugar metabolism, and MAPK signaling pathway - yeast pathways, are shown in Fig. 2.

### Candidate effectors among the 2784 DEGs

One hundred and fifty-nine proteins containing signal peptides were screened among the 2784 proteins encoded by the DEGs using SignalP 4.1. A total of 118 proteins were found by TMHMM 2.0 to lack transmembrane domains. Then, EffectorP 2.0 [22] was used for further clarification, and 54 candidate effectors were ultimately screened. There were 21 upregulated candidate effector genes and 33 downregulated candidate effector genes in THTT compared with THTS. Structural analysis was conducted via a bioinformatics method (Table S5). The protein sizes ranged from 59 to 364 aa. In all, forty-one candidate effector sequences contained more than three cysteines. The predicted 54 effector proteins were analyzed using the Nr database. The results showed that only Unigene22186 had a functional annotation, which was copper/zinc superoxide dismutase (SOD), and the rest were all hypothetical proteins. Domain analysis was performed using Pfam software, and glutaredoxin, DPBB_1, thaumatin, Calc_CGRP_IAPP, MF_alpha_N, TIG, Mtd_N, Omp_AT, Glyco_hydro_7, ConA-like_dom_sf, RNA_pol_Rpb2_3, Kre9/Knh1, CAP, OSTbeta, BAF250_C and Cys domains were found. Perl software was used to search for known motifs, including RxLR in oomycetes, [Y/F/W] xC in powdery mildew and G [I/F/Y][A/L/S/T] R in flax rust. The motif search revealed that 2 effector proteins contained the RxLR motif and that 20 effector proteins contained the [Y/F/W] xC motif; however, no proteins with the G [I/F/Y][A/L/S/T] R motif were found.

### qRT-PCR analysis of 12 DEGs

**Fig. 3:**
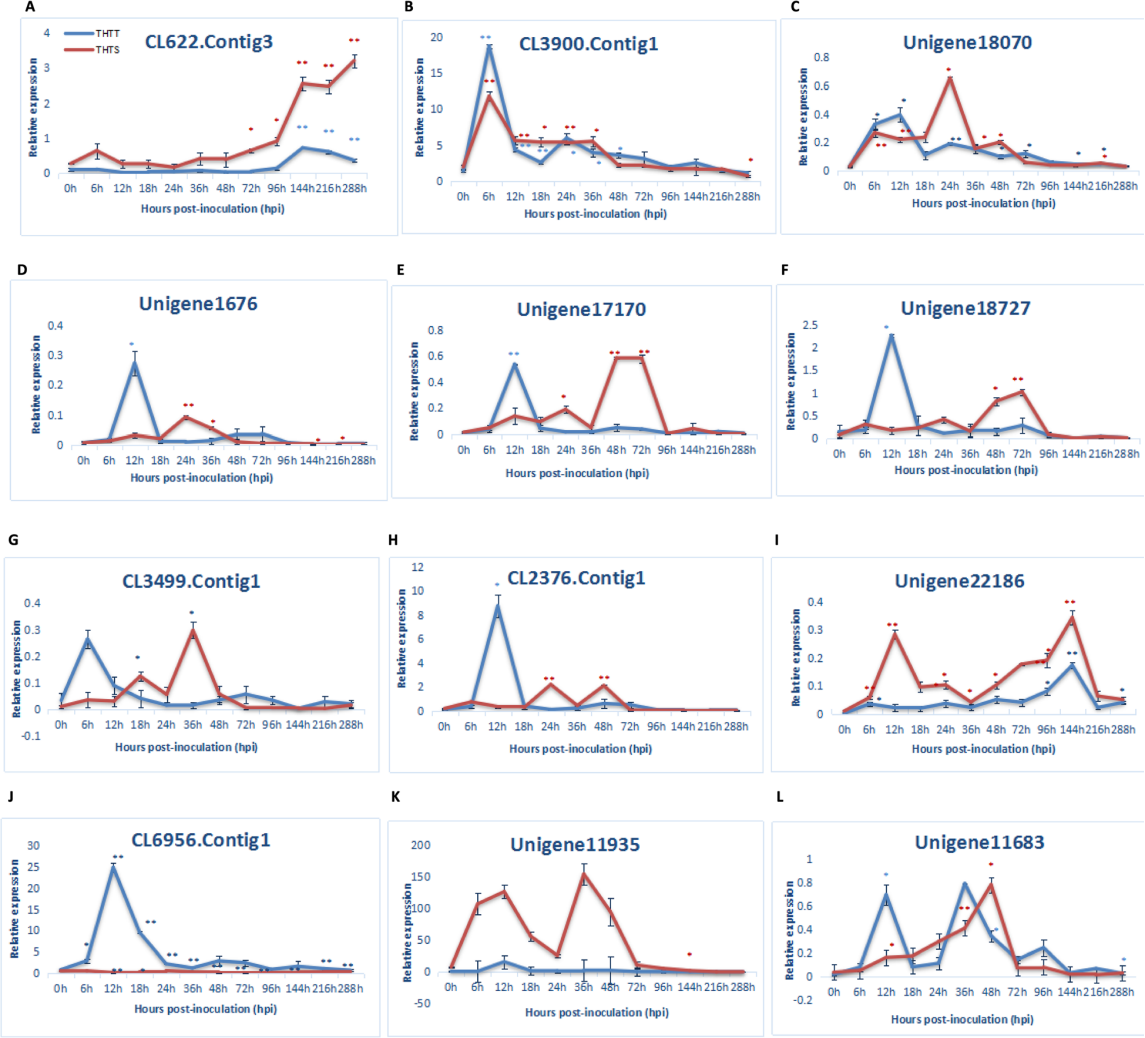
**a-l** Transcriptional profile of genes during the *Pt* pathotypes infection measured by qRT-PCR. Note: P value < 0.05, *; P value < 0.01, **

The RNA-seq results were similar to the qRT-PCR results at 144 hpi. The gene expression trends were related to the formation of structures after rust infection. By 12 hpi, the germ tube, appressorium and substomatal vesicle were clearly formed. Primary hyphae and haustorial mother cells were formed by 18–24 hpi, and secondary hyphae and haustoria were formed by 36 hpi. By 48 hpi, small mycelial masses were formed [23]. To confirm that the trends in gene expression were consistent with the results of RNA-seq and to elucidate the expression characteristics at different time points, we selected twelve important DEGs for real-time fluorescence quantitative analysis (Fig. 3).

Three genes enriched in different KEGG pathways were chosen for analysis. CL622.Contig1 (Fig. 3a) expression peaked at 288 hpi in THTS and at 144 hpi in THTT. CL3900.Contig1 (Fig. 3b) was significantly upregulated at 6 hpi in THTT and THTS (to a significantly greater extent in THTT than in THTS); after 6 hpi, the expression decreased. This gene was annotated as an ATPase. Based on the expression properties, the spores were in the stages of germination and bud tube formation at 6 hpi; therefore, we speculate that CL3900.Contig1 is an energy carrier that participates in these processes. Unigene18070 (Fig. 3c) was annotated to be involved in helicase activity and DNA binding. According to the qRT-PCR results, this gene reached the highest expression level in THTS at 24 hpi, while it reached the highest expression level in THTT at 12 hpi, and the expression level in THTS was higher than that in THTT. Huang et al. [23] observed that *Pt* forms appressoria beginning at 8 hpi. Additionally, haustorial mother cells and primary hyphae develop when *Pt* contacts an epidermal or a mesophyll cell [24]. We suggest that appressorium formation occurred earlier in THTT than in THTS.

According to RNA-seq, Unigene1676 and Unigene17170 were specifically expressed in THTT at 144 hpi, while Unigene18727, CL3499.Contig2 and CL2376.Contig1 were specifically expressed in THTS at 144 hpi. However, according to qRT-PCR, these genes were not specifically expressed at time points other than 144 hpi. The peaks in expression of both Unigene1676 (Fig. 3d) and Unigene17170 (Fig. 3e) occurred at 12 hpi in THTT, earlier than those in THTS. Unigene17170 expression had two peaks at 48 hpi and 72 hpi in THTS. However, Unigene17170 expression peaked at 12 hpi in THTT. These genes were annotated as a ubiquitin (Ub) E3 ligase, a GTPase and an NADPH oxidase. Unigene18727 (Fig. 3f), CL3499.Contig2 (Fig. 3g), and CL2376.Contig1 (Fig. 3h) were expressed earlier in THTT than in THTS, and their expression peaked at 12 hpi in THTT. In contrast, in THTS, Unigene18727 expression peaked at 72 hpi, CL3499.Contig2 expression peaked at 36 hpi, and CL2376.Contig1 expression peaked at 24 hpi and 48 hpi. Unigene18727, CL3499.Contig2 and CL2376.Contig1 were annotated as CYP, ABC transporter and chitinase, respectively.

The expression features of two candidate effectors, Unigene22186 and CL6956.Contig1, were analyzed. The expression trends of Unigene22186 (Fig. 3i), which exhibits SOD activity, were similar in both THTS and THTT, although Unigene22186 expression in THTS was higher than that in THTT. Two peaks were observed at 12 hpi and 144 hpi in THTS, and the highest peak appeared at 144 hpi. In THTT, the expression began to increase at 48 hpi and peaked at 144 hpi. The expression levels of CL6956.Contig1 (Fig. 3j), annotated to have hydrolase activity, were higher in THTT than in THTS. The expression of this gene peaked at 12 hpi in both pathotypes and then gradually decreased.

Two other differentially expressed effectors were also analyzed. The expression of Unigene11935 (Fig. 3k) was very low in THTT at every time point but peaked at 36 hpi in THTS. There was no expression of Unigene11683 (Fig. 3l) in THTT at 144 hpi. However, two peaks were detected at 12 hpi and 36 hpi. Unigene11683 expression peaked at 48 hpi in THTS. Therefore, Unigene11935, which was expressed only in THTS, was a specifically expressed DEG.

### Unigene23118 and Unigene17565 could inhibit programmed cell death (PCD) induced by BAX in *N. benthamiana*

**Fig. 4:**
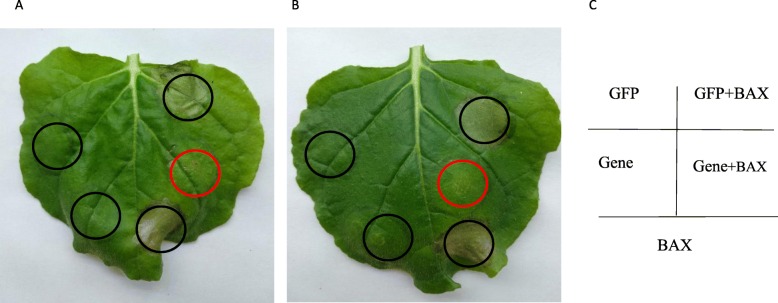
The candidate effector proteins that can inhibit the cell death triggered by BAX. **a** and **b** represent Unigene23118 and Unigene17565, respectively. **c** is the schematic of injection

To test whether the effector proteins could inhibit PCD, the recombinant plasmids, BAX, and negative control-eGFP were injected into *N. benthamiana.* There was clearly no homologous recombination (HR) at the sites where the vector, Unigene23118 and Unigene17565 were injected alone. Necrosis occurred at the sites where BAX was injected alone and where BAX and the vector were injected together. However, there was no necrosis at the sites where BAX and either Unigene23118 (Fig. 4a) or Unigene17565 (Fig. 4b) were injected together, indicating that these genes inhibited BAX action, preventing PCD from being induced. Thus, we can conclude that Unigene23118 and Unigene17565 have effector functions.

## Discussion

### THTT and THTS exhibited complicated expression patterns when interacting with Thatcher at 144 hpi

The pathotypes exhibited many differences during the interaction with Thatcher at 144 hpi. A total of 1076 of the 10,483 upregulated genes in THTT compared to THTS were significantly upregulated, while 1708 of the 10,689 downregulated genes were significantly downregulated. We aligned 566 significantly upregulated genes and 1225 downregulated genes to KEGG and GO. Of the 2784 genes with significantly different expression, 100 genes were present in the NCBI Nr database. Seventy-eight of these genes were significantly upregulated in THTT compared to THTS, while 22 were significantly downregulated. GO analysis was performed to classify the DEGs into 3 major biological categories. These genes belonged to 19 classes in the biological process category, 9 classes in the cellular component category and 12 classes in the molecular function category.

According to the RNA-seq library, CL622.Contig3 was annotated as mannosyl-oligosaccharide glucosidase (MOGS). OsMOGS was involved in N-glycan synthesis in rice and played important roles in establishing and maintaining the growth of auxin and the growth and development of the root system [25]. Therefore, we speculate that this gene participates in epithelial cell development and mycelial growth. Unigene1676 was annotated as a Ub E3 ligase. E3 Ub ligases transfer Ub to one or more Lys residues in a substrate by linking the C-terminal Gly of Ub with the Lys residue of the target protein (and/or a Lys of the Ub itself). Ub E3 ligases are closely related to cell cycle regulation, tumorigenesis, cell proliferation, apoptosis, signal transduction, cell growth, cell immunity, inflammation and the regulation of DNA replication and repair. The F-box protein (SCF) E3 ligases are the largest E3 gene family, of which the F-box protein is the key component determining substrate specificity. One study has revealed that a deletion mutant of GrrA, an F-box protein in *Aspergillus nidulans,* is unable to produce mature ascospores because of a block in meiosis [26].

GTPases (singular GTPase) are a large family of hydrolase enzymes that can bind and hydrolyze GTP. GTP binding and hydrolysis take place in the highly conserved G domain common to all GTPases. Zhang et al. [27] verified the functions of all six Rho GTPases in *Fusarium graminearum* by constructing deletion vectors. The mutants △Fgrac1, △Fgcdc42 and △Fgrho4 exhibited drastic reductions in growth rate, and △Fgrho4 lacked aerial hyphae. △Fgrho2 and △Fgrho3 exhibited less drastic growth rates on CM plates than other mutants. FgRho1 was found to be essential for fungal survival. FgRho2, FgRho4, FgCdc42 and FgRac1 were found to be involved in sexual development and pathogenesis, while FgRho2 and FgRho4 were both found to be associated with cell wall integrity. Only FgRho4 showed a role in nuclear division and septum formation. In addition, knockdown (KD) of TaRab7 has been found to enhance the susceptibility of wheat cultivar Suwon 11 to the avirulent race CYR23, which implies that TaRab7 plays important roles in the early stage of wheat stripe rust fungus interaction and in stress tolerance [28]. In our research, Unigene17170 expression peaked at 12 hpi in THTT and peaked at 48–72 hpi in THTS when forming an appressorium; thus, this gene may be involved in the formation of hyphae.

### qRT-PCR revealed more detailed differential expression patterns between THTT and THTS at the early stage

The qRT-PCR patterns were almost the same as the RNA-seq patterns at 144 hpi. However, there were more differential expression patterns at the early stage than at 144 hpi. Twelve tested genes had obvious peaks in expression before 144 hpi (Fig. 3).

The expression of 9 genes expressed earlier in THTT than in THTS. CL3900.Contig1 expression peaked at 6 hpi in both THTT and THTS. CL3499.Contig1 expression peaked at 6 hpi in THTT and at 36 hpi in THTS.

CL6956.Contig1 expression peaked at 12 hpi in THTT and was expressed at a much higher level in THTT than in THTS. Unigene18070 expression peaked at 12 hpi in THTT and at 24 hpi in THTS. CL2376.Contig1 expression peaked at 12 hpi in THTT and at 24 hpi and 48 hpi in THTS. In addition, Unigene17170 expression peaked at 12 hpi in THTT, but it peaked at 48 hpi in THTS at a higher level than that in THTT. Unigene1676 expression in THTT peaked at 12 hpi at a level three times that in THTS. Unigene11683 expression exhibited two peaks at 12 hpi and 36 hpi in THTT and one peak at 48 hpi in THTS. Unigene18727 was most highly expressed at 12 hpi in THTT.

The expression of 4 genes appeared earlier in THTS than in THTT. CL622.Contig3 was expressed at 6 hpi, and its levels in THTT were higher than those in THTS. Unigene22186 expression had two peaks at 12 hpi and 144 hpi in THTS and one peak at 144 hpi in THTT. Unigene11935 expression peaked at 12 hpi and 36 hpi in THTS at more than 100 times the level of expression in THTT. Unigene14763 had almost the same expression pattern in the two races.

Unigene18727 was found to have a conserved domain of CYP 52A6. CYP53 family members play key roles in fungal colonization of plant material by detoxifying antifungal compounds released by plants or generated during plant material degradation. Moreover, CYP53 family members play roles in the generation of a secondary metabolite, veratryl alcohol, that is crucial in the degradation of the plant cell wall component lignin [29]. The expression of Unigene18727 peaked in THTT at 12 hpi; in THTS, it gradually increased and peaked at 72 hpi. However, it was hardly expressed at later stages. Given this information and the cytochrome P450 function of the gene, we hypothesize that this gene aids in host infection. This gene was expressed earlier in THTT than in THTS.

CL2376.Contig1 is a member of the ABC transporter C family. ABC transporters belong to a large and ancient protein family. Energy produced by ATP binding and hydrolysis is used for ABC transporter participation in substrate transport processes, such as the RNA translation and transmembrane processes required for DNA repair. Although the mechanism is unclear, these transporters play important roles in enhancing the ability of pathogens to resist adverse external environments. CL2376.Contig1 expression had two peaks in THTS (at 24 hpi and 48 hpi) and only one peak in THTT (at 12 hpi), and the expression level was significantly higher in THTT than in THTS; thus, this gene may play a role in the pathogenic process.

Chitinase can degrade most fungal cell walls and prevent or interrupt fungal infection, colonization and expansion in plants. Chitin deacetylase (CDA) is a chitinase. The enhanced CDA activity during appressorium formation in Uromyces and the appressorium formation failure in polycarbonate artificial culture medium exhibited by Magnaporthe oryzae CDA deletion mutants both indicate that CDA is involved in the formation of appressorium [30]. Furthermore, CDA has a promoting effect on the formation of fungal fruiting bodies. CDA isolated from the basidiomycetes of Flammulina velutipes is specifically expressed during the fruiting stage [31]. CL3499.Contig2 was predicted to encode CDA. The expression of this gene increased until peaking at 6 hpi in THTT and at 72 hpi in THTS. This gene was expressed earlier in THTT than in THTS. Therefore, we speculate that the CL3499.Contig2_Tc15_2 gene may play a key role in pathogenic infection of leaf rusts.

The effector Unigene22186 has SOD activity. MnSOD plays a role in organisms facing exogenous oxidative reactive oxygen species (ROS) and especially superoxide overproduction [32]. The role of MnSOD was deduced from the increase in SOD2 expression in *S. pombe* after exposure to menadione. Furthermore, SOD2 mutants were more sensitive than wild-type organisms to menadione and to plumbagin, a menadione derivative. SOD2 plays important roles in the virulence of both *C. neoformans* var. *gattii* and *C. neoformans* var. *grubii*, depending on the route of infection [33, 34]. Since defense against ROS is a determinant of pathogenicity, the evolutionary history of MnSOD and the pathophysiological roles of MnSOD in invasive or allergic mycoses support the hypothesis that pathogenicity has emerged multiple times within fungi. MnSOD has been used for taxonomic and evolutionary data analyses [35]. This gene was expressed in THTT, and its expression increased beginning at 6 hpi and peaked at 144 hpi; however, its expression in THTS exhibited two peaks at 12 hpi and 144 hpi, and the expression levels were higher in THTS than in THTT. We hypothesize that Unigene22186 is a disease-related gene.

Many factors that lead to differences between the two strains. To explore the reasons for the toxicity differences between these two strains, we will focus on the genes with specific expression profiles in future experiments.

### Effectors secreted by THTT and THTS when interacted with Thatcher have different expression patterns

The functional domains were obtained by Pfam to clarify the biological functions of the 54 candidate secreted proteins (Table S5) and included domains are associated with the rare lipoprotein A (RlpA)-like domain superfamily, the osmotin or thaumatin superfamily, the glycoside hydrolase family 7, the concanavalin A-like lectin, the Kre9/Knh1 family, and so on.

The candidate effector Unigene15605 was upregulated in THTS. The Kre9/Knh1 family is related to cell wall organization in fungi. Gilbert et al. [36] found that the H99 deletion mutants kre5Δ and kre6Δskn1Δ contain less β-1,6-glucan, which is a component of the cell wall, than wild-type organisms and grew slowly with an aberrant morphology. Moreover, these two mutants exhibited alterations in cell wall chitosan and the exopolysaccharide capsule, a primary cryptococcal virulence determinant.

Amino acids 25–66 of Unigene11683 were similar to a concanavalin A-like lectin family sequence. Previous sequence analyses have shown that the N-terminal regions of BEACH proteins share similarities with the concanavalin A (ConA)-like lectin superfamily, and members of the BEACH family are generally defined as trafficking regulators of vesicles, which are transport carriers involved in the process of protein secretion [37]. However, amino acids 21–69 of Unigene11683 were aligned with glycoside hydrolase family 7. Mutants of cellulases which can hydrolyze crystalline cellulose were constructed by KD to permeate the host epidermis, to clarify that the cellulases belonged to glycosyl hydrolase (GH) families 6 and 7. The results showed that the transcript levels of the target genes and cellulase activity were considerably reduced in the KD mutants, and the KD mutants resulted in fewer lesions and less penetration and fewer infected cells than the parent strain [38].

Fifty-four candidate differentially expressed effectors were predicted in this study, and the expression profiles of 4 candidate effectors were obtained. Ultimately, two candidate effectors were identified. These results lay the foundation for further study of the pathogenic differences among *Pt* pathotypes at the molecular level and reveal the interaction mechanisms. However, this study was based mainly on bioinformatics, macroscopic screening and qRT-PCR technology; thus, the specific DEGs should be further investigated. Other members of our experimental group will perform studies using gene silencing techniques and will inoculate tobacco with the pathogenic genes to verify the functions of the genes.

## Conclusion

According to the transcriptome sequencing analysis between two different isolates, it was found that although the pathogenicity of the two strains only differed in *Lr18*, *Lr36*, and *Lr44*, there were 2784 DEGs. In addition, few genes of the 12 DEGs expressed similarly in two isolates. This finding indicates that although the interaction between leaf rust and wheat is based on the gene-to-gene hypothesis in the pathogenic process, instead of merely a pair of genes, a large number of genes participate in the regulation.

The study found that there are 2 types of conserved motifs that have been reported among 54 candidate effector proteins. Two effector proteins contain the RxLR motif and 20 effector proteins contain the [Y/F/W] xC motif, which proves that the effectors of *Pt* are highly diverse; however, most of them are specific to wheat leaf rust.

The results obtained in this study might lay a solid foundation for future studies on clarifying the mechanisms of pathogenicity differences among Chinese isolates and elucidating the pathogenic mechanism of *Pt*. The pathogenesis of wheat leaf rust is very complex, although the pathotypes are very similar. The virulence results in significant changes at the molecular level in only a small number of genes according to the virulence formula. Therefore, exploring the pathogenic mechanisms is a long-term, arduous and crucial task. Consistent exploration of wheat leaf rust effectors, elucidation of their functions in pathogenesis and location and targeting of their receptors in plants will be indispensable. In future studies, we will focus on several effectors that are differentially expressed between THTT and THTS to explore avirulent genes. Overall, this study provides a basis for understanding the molecular mechanisms of wheat resistance to persistent diseases, the control of wheat rust, and the pathogenic mechanisms of specific parasitic fungi.

## Supplementary information

**Additional file 1: Table S1.** The primers for qRT-PCR and the annotation of chosen genes for q-PCR **Table S2.** The primers for transient transformation on *Nicotiana benthamiana***Table S3.** Forty-five specific expressed genes in THTT at 144 hpi **Table S4.** Twenty six specific expressed genes in THTS at 144 hpi **Table S5.** Sequence analysis of canddate effectors **Table S6.** The expression of candidate effectors in THTS and THTT

## Data Availability

The sequencing data were submitted to the Sequence Read Archive (Accession number PRJNA613154; www.ncbi.nlm.nih.gov/Traces/study/?acc=PRJNA613154) and Transcriptome Shotgun Assembly (Accession Number GIKZ00000000) in NCBI.

## Abbreviations

*Pt*: *Puccinia triticina,* Eriks
hpi: Hours post inoculation
DEGs: Differentially expressed genes
SSH: Suppression subtractive hybridization
2D-PAGE: Two-dimensional polyacrylamide gel electrophoresis
EST: Expressed sequence tag
*Mlp*: *Melampsora larici-populina*
*Pgt*: *Puccinia graminis* f. sp. *tritici*
RPKM: Reads per Kb per million reads
FDR: False discovery rate
GO: Gene Ontology
KEGG: Kyoto Encyclopedia of Genes and Genomes
Nr: Nonredundant
ATPase: Adenosine triphosphatase
SOD: Superoxide dismutase
Ub: Ubiquitin
CYP: cytochrome P450
ABC: ATP binding cassette
PCR: Polymerase chain reaction
qPCR: Quantitative PCR
PCD: Programmed cell death
MOGS: Mannosyl-oligosaccharide glucosidase
KD: Knock-down
CDA: Chitin deacetylase
ROS: Reactive oxygen species
ConA: Concanavalin A
GH: Glycosyl hydrolase
