# Supplementary material for: Puccinia triticina pathotypes THTT and THTS display complex transcript profiles on wheat cultivar Thatcher
Source: BMC Genet. 2020 Apr 28;21:48. doi: 10.1186/s12863-020-00851-5 (PMC7189582; doi:10.1186/s12863-020-00851-5)
Supplement: Supplementary file 1 — Additional file 1: Table S1. The primers for qRT-PCR and the annotation of chosen genes for q-PCR Table S2. The primers for transient transformation on Nicotiana benthamianaTable S3. Forty-five specific expressed genes in THTT at 144 hpi Table S4. Twenty six specific expressed genes in THTS at 144 hpi Table S5. Sequence analysis of canddate effectors Table S6. The expression of candidate effectors in THTS and THTT [file 12863_2020_851_MOESM1_ESM.docx]

Table S1 The primers for qRT-PCR and the annotation of chosen genes for q-PCR

| GeneID | Primer name | Primer sequence（5'-3') | Sequence length/bp | Gene length/bp | THTS  RPKM | THTT  RPKM | Annotation |
| --- | --- | --- | --- | --- | --- | --- | --- |
| CL622.Contig3_Tc15_2 | RT622 | F-CCGAGCTGTACGAGGAACTG | 20 | 2695 | 37.80 | 14.14 | mannosyl-oligosaccharide glucosidase activity(GO) |
|  |  | R-CGCCGGTGATAGGGTCATAC | 20 |  |  |  |  |
| CL3900.Contig1_Tc15_2 | RT3900 | F-CTTTTGGGAACGGCACGAAG | 20 | 1608 | 0.79 | 0.31 | Adenosinetriphosphatase  (nt) |
|  |  | R-TGCATGGGTAACTTGACGCT | 20 |  |  |  |  |
| Unigene18070_Tc15_2 | RT18070 | F-TACTGGGGCGACTCAGTGTA | 20 | 2660 | 58.72 | 26.77 | helicase activity;DNA binding;ATP binding(GO) |
|  |  | R-CTTCGAGCCGGTTAGGTGAG | 20 |  |  |  |  |
| Unigene1676_Tc15_2 | RT1676 | F-GATAAAGACGGTGCACCTCC | 20 | 274 | 0.01 | 29.28 | E3 ubiquitin-protein ligase(ko) |
|  |  | R-CGTCGTCATCCTCTTCCTCA | 20 |  |  |  |  |
| Unigene17170_Tc15_2 | RT17170 | F-CGGCAAGGACTCCATCTCC | 19 | 385 | 0.01 | 40.38 | glucose repressible protein 2(KEGG) |
|  |  | R-CCCTCGTGCTTCTTCTCGT | 19 |  |  |  |  |
| Unigene18727_Tc15_2 | RT18727 | F-TTCTCCTTGATCTCCGCCTC | 20 | 574 | 12.47 | 0.01 | Cytochrome P450 52A6(nr) |
|  |  | R-CTGGACAGGGAGATCAGGC | 19 |  |  |  |  |
| CL3499.Contig2_Tc15_2 | RT3499 | F-GTGCTCGTGGTTGGGTGG | 18 | 673 | 9.38 | 0.01 | Flocculation protein |
|  |  | R-ACTGCATGTCATACCACTGG | 20 |  |  |  |  |
| CL2376.Contig1_Tc15_2 | RT2376 | F-CAAACAGGAACCGTGCAAGA | 20 | 354 | 24.97 | 0.01 | ATP-binding cassette transporter 2(GO) |
|  |  | R-GTCCTTGACCAACGAGCAC | 19 |  |  |  |  |
| Unigene22186_Tc15_2 | RT22186 | F-TTGCAATCCGTCATGGCAAC | 20 | 992 | 199.46 | 41.96 | metal ion binding; superoxide dismutase activity(GO) |
|  |  | R-TTCGCGCCATGAATGACAAC | 20 |  |  |  |  |
| CL6956.Contig1_Tc15_2 | RT6956 | F-TGTTGCTTGGAGAGTACGGC | 20 | 1292 | 196.15 | 1501.69 | -- |
|  |  | R-CAAGTCTTGGTGTTTCCGCC | 20 |  |  |  |  |

| Unigene11683_Tc15_2 | RT11683 | F-GACGAGTCTGTGGTGTCTCT | 20 | 289 | 24.76 | 0.01 | -- |
| --- | --- | --- | --- | --- | --- | --- | --- |
|  |  | R-CGTGATGCTCAGTCGCTTAT | 20 |  |  |  |  |
| Unigene11935_Tc15_2 | RT11935 | F-CGCTCAGAAAACCAAACCTC | 20 | 427 | 20.70 | 0.01 | -- |
|  |  | R-ATTGTGACGCCAGCCTTAGT | 20 |  |  |  |  |

Table S2 The primers for transient transformation on *Nicotiana benthamiana*

| Primer name | Primer sequence（5'-3') | Sequence length/bp |
| --- | --- | --- |
| U17565-F | ATCGATGATGTATTCGTGTGCCCC | 24 |
| U17565-R | CCCGGGATGGGTGGTCAGTATATT | 24 |
| U23118-F | ATCGATAATTGGGACCCCGCAACG | 24 |
| U23118-R | CCCGGGATCACTGTTGTCAGGAGA | 24 |

Table S3 Forty-five specific expressed genes in THTT at 144 hpi

| Gene ID | Deduced character and function | Gene ID | Deduced character and function | |
| --- | --- | --- | --- | --- |
| CL6859.  Contig2_Tc15_2 | Hypothetical protein PGTG_07569 | CL5715.Contig2_Tc15_2 | | Hypothetical protein CERSUDRAFT_115801  (*Ceriporiopsis subvermispora*) |
| Unigene12464  _Tc15_2 | Transcription initiation factor TFIIIB(KO);Hypothetical protein RO3G_11938 (*Rhizopus delemar*) | Unigene15745  _Tc15_2 | histone-lysine N-methyltransferase (KO);Hypothetical protein DOTSEDRAFT_40277  （*Clostridium perfringens*） | |
| Unigene22345  _Tc15_2 | Hypothetical protein PGTG_10413 | Unigene25145  _Tc15_2 | Hypothetical protein PGTG_18554 | |
| Unigene7211  _Tc15_2 | Hypothetical protein PGTG_19708 | Unigene2529  _Tc15_2 | Hypothetical protein PGTG_00998 | |
| CL5307.Contig2  _Tc15_2 | Hypothetical protein PGTG_19579 | Unigene11430  _Tc15_2 | Hypothetical protein PGTG_03295 | |
| CL560.Contig1  _Tc15_2 | Hypothetical protein PGTG_13958 | Unigene7449  _Tc15_2 | Hypothetical protein PGTG_05143 | |
| CL7155.Contig1  _Tc15_2 | Glucoamylas(KO);Hypothetical protein PGTG_08062 | Unigene9988_  Tc15_2 | Putative uncharacterized protein YHR217C(*S cerevisiae*) | |
| Unigene26456  _Tc15_2 | Hypothetical protein PGTG_13885 | Unigene25109  _Tc15_2 | Hypothetical protein PGTG_15083 | |
| CL4985.Contig1  _Tc15_2 | Asparagine synthase (glutamine-hydrolysing)  (*Exophiala dermatitidis*)(KO) | CL3109.Contig3_Tc15_2 | Hypothetical protein PGTG_18680 | |
| Unigene24409  _Tc15_2 | Predicted protein (*Pyrenophora tritici-repentis*] | CL1464.Contig1_Tc15_2 | D-amino-acid oxidase(*Rhodosporidium toruloides*)(KO) | |
| Unigene17702  _Tc15_2 | Large subunit ribosomal protein L41e(*Saccharomyces cerevisiae*) | Unigene26432_Tc15_2 | Hypothetical protein PGTG_15563 | |
| Unigene11040  _Tc15_2 | substrate-specific transmembrane transporter activity(GO);Hypothetical protein MELLADRAFT_71916  (*Melampsora larici-populina*) | Unigene17170  _Tc15_2 | Glucose repressible protein gene(*Xanthophyllomyces dendrorhous*)(Nt)；Histone-lysine N-methyltransferase SETD1(ko); Small GTPase mediated signal transduction(GO) | |
| Unigene24199_Tc15_2 | Hypothetical protein PGTG_07304 | Unigene25090  _Tc15_2 | Hypothetical protein PGTG_03348 | |
| CL5606.Contig1  _Tc15_2 | Hypothetical protein PGTG_07990， | CL1408.Contig1_Tc15_2 | Hypothetical protein PGTG_04420， | |
| Unigene7326_Tc15_2 | Hypothetical protein PGTG_05143， | Unigene7450  _Tc15_2 | Hypothetical protein PGTG_05143 | |
| Unigene11422  _Tc15_2 | Nucleolar complex protein 2(S. cerevisiae) | Unigene23304  _Tc15_2 | alpha-amylase(KO);Unnamed protein product(*Aspergillus oryzae*) | |
| Unigene28255  _Tc15_2 | Hypothetical protein PGTG_03418 | Unigene10946  _Tc15_2 | Hypothetical protein PGTG_19367 | |
| CL4323.Contig1  _Tc15_2 | Hypothetical protein PGTG_05160 | CL4476.Contig1_Tc15_2 | Chitinase(KO); Hypothetical protein TRIVIDRAFT_225056  ( *Trichoderma virens*) | |
| Unigene23386  _Tc15_2 | Hypothetical protein AURDEDRAFT_149120 (*Auricularia delicate*) | Unigene11154  _Tc15_2 | Hypothetical protein PGTG_15082(Nr);Nucleic acid binding(GO) | |
| Unigene1676  _Tc15_2 | E3 ubiquitin-protein ligase TRIP12 | Unigene10945  _Tc15_2 | Hypothetical protein PGTG_12382 | |
| Unigene4619  _Tc15_2 | large subunit ribosomal protein L41e (*Ashbya gossypii*) | CL420.Contig2  _Tc15_2 | Hypothetical protein PGTG_19367 | |
| CL6460.Contig1  _Tc15_2 | Asparagine synthase (glutamine-hydrolyzing)  (*Rhizopus delemar*) | Unigene14298  _Tc15_2 | Hypothetical protein PGTG_10756 | |

Table S4 Twenty six specific expressed genes in THTS at 144 hpi

| Gene ID | Deduced character and function | Gene ID | Deduced character and function |
| --- | --- | --- | --- |
| Unigene31407  _Tc15_2 | Nitrate reductase (NADPH)(*Ustilago maydis*)(KO) | Unigene7678  _Tc15_2 | Hypothetical protein PGTG_17070 |
| Unigene3535  _Tc15_2 | H+-transporting ATPase  (*P. graminis* f. sp. tritici)(KO) | Unigene24030  _Tc15_2 | Hypothetical protein PGTG_19367 |
| Unigene31515  _Tc15_2 | Cytochrome-b5 reductase(*Fomitiporia mediterranea*)(KO) | CL5794.Contig1  _Tc15_2 | Hypothetical protein AOL_s00110g124  (*Arthrobotrys oligospora*) |
| Unigene15719  _Tc15_2 | GTPase activity(GO); Hypothetical protein PGTG_14941 | Unigene10373  _Tc15_2 | Hypothetical protein PGTG_02401 |
| Unigene2587  2_Tc15_2 | —— | Unigene18727  _Tc15_2 | Cytochrome P450  (*Candida tropicalis*) |
| CL2376.Contig1  _Tc15_2 | Putative ABC transport system (Batrachochytrium dendrobatidis)(KO) | CL1500.Contig2  _Tc15_2 | L:anosterol synthase  (Schizosaccharomyces pombe) |
| Unigene11683  _Tc15_2 | Hypothetical protein PGTG_17415 | Unigene20992  _Tc15_2 | putative replicase readthrough protein(Eutypa lata) |
| Unigene5804  _Tc15_2 | Hypothetical protein PGTG_01823 | CL3499.Contig2  _Tc15_2 | GTPase activator activity(GO); Hypothetical protein PGTG_05787 |
| Unigene19721  _Tc15_2 | Terpenoid cylase  (*P. graminis* f. sp. tritici) | CL5691.Contig1  _Tc15_2 | cell wall integrity and stress response component  (*S. cerevisiae*) |
| Unigene3471  _Tc15_2 | Hypothetica protein PGTG_01823 | Unigene337  _Tc15_2 | Hypothetical protein CMQ_2906  (*Grosmannia clavigera*) |
| Unigene3938  _Tc15_2 | Hypothetical protein PGTG_07937 | Unigene11388  _Tc15_2 | uncharacterized protein CPUR_01692  (*Claviceps purpurea*) |
| Unigene11935  _Tc15_2 | Hypothetical protein PGTG_03017 | Unigene20989  _Tc15_2 | Chitinase-like protein  (S. pombe)(KO) |
| Unigene3933  _Tc15_2 | Hypothetical protein PGTG_15242 | CL4245.Contig1  _Tc15_2 | Lon-like ATP-dependent protease（*P. graminis* f. sp. *Tritici*）(KO) |

Table S5 Sequence analysis of canddate effectors

| Gene | Size(aa) | Cys | Best hit in NCBI database | E-value | Pfam motif | Domain |
| --- | --- | --- | --- | --- | --- | --- |
| CL1243.Contig1_Tc15_2 | 91 | 7 | hypothetical protein PGTG_21829 | 1.00E-09 | NO | FxC |
| CL1492.Contig1_Tc15_2 | 214 | 6 | hypothetical protein PGTG_18959 | 1.00E-47 | NO | NO |
| CL2349.Contig2_Tc15_2 | 136 | 4 | hypothetical protein PGTG_21687 | 2.00E-28 | Glutaredoxin | NO |
| CL4021.Contig2_Tc15_2 | 138 | 4 | hypothetical protein PGTG_00796 | 1.00E-06 | NO | NO |
| CL4576.Contig2_Tc15_2 | 195 | 8 | hypothetical protein PGTG_02614 | 9.00E-96 | NO | WxC |
| CL4624.Contig2_Tc15_2 | 162 | 4 | hypothetical protein PGTG_18961 | 6.00E-45 | NO | NO |
| CL5885.Contig1_Tc15_2 | 113 | 4 | hypothetical protein PGTG_01373 | 8.00E-41 | NO | NO |
| CL6671.Contig1_Tc15_2 | 132 | 4 | hypothetical protein PGTG_18777 | 1.00E-56 | DPBB_1 | NO |
| CL6956.Contig1_Tc15_2 | 183 | 8 | hypothetical protein PGTG_19648 | 9.00E-93 | Thaumatin | NO |
| Unigene683_Tc15_2 | 114 | 5 | hypothetical protein PGTG_14433 | 1.00E-11 | NO | NO |
| Unigene2822_Tc15_2 | 105 | 5 | hypothetical protein PGTG_13411 | 1.00E-09 | NO | [Y/F]xC |
| Unigene3256_Tc15_2 | 89 | 0 | hypothetical protein PGTG_14348 | 3.00E-19 | Calc_CGRP_IAPP | NO |
| Unigene3561_Tc15_2 | 132 | 6 | hypothetical protein PGTG_02164 | 1.00E-46 | MF_alpha_N | NO |
| Unigene3795_Tc15_2 | 113 | 6 | hypothetical protein PGTG_13411 | 2.00E-11 | NO | NO |
| Unigene4156_Tc15_2 | 150 | 6 | hypothetical protein PGTG_19647 | 2.00E-66 | TIG | RxLR |
| Unigene4204_Tc15_2 | 120 | 10 | hypothetical protein PGTG_14245 | 1.00E-26 | NO | [Y/F]xC |
| Unigene4298_Tc15_2 | 253 | 17 | hypothetical protein PGTG_18309 | 2.00E-146 | Thaumatin | WxC |
| Unigene4450_Tc15_2 | 211 | 6 | hypothetical protein PGTG_18959 | 4.00E-48 | NO | YxC |
| Unigene4469_Tc15_2 | 87 | 8 | hypothetical protein PGTG_09318 | 5.00E-26 | NO | WxC |
| Unigene4609_Tc15_2 | 164 | 13 | hypothetical protein PGTG_17910 | 2.00E-68 | NO | FxC |
| Unigene4712_Tc15_2 | 200 | 14 | hypothetical protein PGTG_17549 | 2.00E-62 | NO | YxC |
| Unigene5013_Tc15_2 | 123 | 3 | hypothetical protein PGTG_16240 | 7.00E-50 | NO | NO |
| Unigene6906_Tc15_2 | 151 | 10 | hypothetical protein PGTG_13503 | 1.00E-23 | NO | YxC |
| Unigene7571_Tc15_2 | 193 | 8 | hypothetical protein PGTG_15673 | 1.00E-83 | NO | NO |
| Unigene8006_Tc15_2 | 177 | 11 | hypothetical protein PGTG_16570 | 1.00E-79 | NO | FxC |
| Unigene8115_Tc15_2 | 186 | 3 | hypothetical protein PGTG_00495 | 8.00E-69 | Mtd_N | NO |
| Unigene8591_Tc15_2 | 228 | 4 | hypothetical protein PGTG_06328 | 3.00E-98 | NO | RxLR |
| Unigene8845_Tc15_2 | 221 | 3 | hypothetical protein PGTG_14974 | 1.00E-106 | NO | FxC |
| Unigene9122_Tc15_2 | 59 | 3 | hypothetical protein PGTG_11681 | 1.00E-12 | NO | NO |
| Unigene9803_Tc15_2 | 269 | 8 | hypothetical protein PGTG_10789 | 1.00E-142 | NO | NO |
| Unigene9857_Tc15_2 | 185 | 1 | hypothetical protein PGTG_12528 | 6.00E-53 | Omp_AT | NO |
| Unigene10936_Tc15_2 | 133 | 12 | hypothetical protein PGTG_12153 | 2.00E-34 | NO | WxC |
| Unigene11651_Tc15_2 | 141 | 11 | hypothetical protein PGTG_03185 | 9.00E-65 | NO | [Y/F]xC |
| Unigene11683_Tc15_2 | 69 | 3 | hypothetical protein PGTG_17415 | 1.00E-16 | Glyco_hydro_7  ConA-like_dom_sf | NO |
| Unigene11935_Tc15_2 | 108 | 0 | hypothetical protein PGTG_03017 | 7.00E-29 | NO | RxLR |
| Unigene12071_Tc15_2 | 78 | 3 | hypothetical protein PGTG_07911 | 4.00E-21 | RNA_pol_Rpb2_3 | NO |
| Unigene12860_Tc15_2 | 252 | 14 | hypothetical protein PGTG_00898 | 6.00E-132 | NO | NO |
| Unigene12905_Tc15_2 | 117 | 7 | hypothetical protein PGTG_08470 | 5.00E-40 | NO | FxC |
| Unigene13539_Tc15_2 | 212 | 8 | hypothetical protein PGTG_06969 | 2.00E-82 | NO | NO |
| Unigene15092_Tc15_2 | 252 | 16 | hypothetical protein PGTG_18309 | 1.00E-131 | Thaumatin | WxC |
| Unigene15605_Tc15_2 | 132 | 2 | hypothetical protein PGTG_12305 | 5.00E-52 | Kre9/Knh1 family | NO |
| Unigene16387_Tc15_2 | 203 | 9 | hypothetical protein PGTG_06854 | 1.00E-45 | NO | YxC |
| Unigene17187_Tc15_2 | 177 | 12 | hypothetical protein PGTG_17547 | 1.00E-76 | NO | YxC |
| Unigene17565_Tc15_2 | 127 | 5 | hypothetical protein PGTG_02156 | 4.00E-08 | NO | NO |
| Unigene17799_Tc15_2 | 80 | 2 | hypothetical protein PGTG_04987 | 6.00E-10 | NO | YxC |
| Unigene18229_Tc15_2 | 191 | 12 | hypothetical protein PGTG_08705 | 2.00E-17 | NO | FxC |
| Unigene18358_Tc15_2 | 181 | 2 | hypothetical protein PGTG_17020 | 2.00E-30 | NO | NO |
| Unigene21130_Tc15_2 | 237 | 1 | hypothetical protein PGTG_08644 | 8.00E-50 | NO | NO |
| Unigene22186_Tc15_2 | 186 | 5 | copper/zinc superoxide dismutase | 1.00E-79 | NO | NO |
| Unigene22680_Tc15_2 | 201 | 1 | hypothetical protein PGTG_18744 | 4.00E-70 | CAP/OSTbeta | NO |
| Unigene22930_Tc15_2 | 364 | 22 | hypothetical protein PGTG_09406 | 1.00E-142 | NO | NO |
| Unigene23118_Tc15_2 | 228 | 6 | hypothetical protein PGTG_15623 | 3.00E-111 | NO | NO |
| Unigene23402_Tc15_2 | 151 | 5 | hypothetical protein PGTG_02197 | 3.00E-42 | BAF250_C/Cys_knot | NO |
| Unigene23624_Tc15_2 | 165 | 4 | hypothetical protein PGTG_18031 | 1.00E-28 | NO | NO |

Table S6 The expression of candidate effectors in THTS and THTT

| Gene ID | RPKM in THTS | RPKM of THTT | Gene ID | RPKM in THTS | RPKM in THTT |
| --- | --- | --- | --- | --- | --- |
| CL1243.Contig1_Tc15_2 | 408.3 | 170.2 | Unigene8845_Tc15_2 | 23.0 | 3.6 |
| CL1492.Contig1_Tc15_2 | 1578.8 | 315.2 | Unigene9122_Tc15_2 | 89.0 | 180.6 |
| CL2349.Contig2_Tc15_2 | \| 165.4 \| 25.07350297 \| \| --- \| --- \| | 25.1 | Unigene9803_Tc15_2 | 345.2 | 114.6 |
| CL4021.Contig2_Tc15_2 | 3351.4 | 740.0 | Unigene9857_Tc15_2 | 37.8 | 270.8 |
| CL4576.Contig2_Tc15_2 | 31.1 | 65.7 | Unigene10936_Tc15_2 | 147.8 | 33.3 |
| CL4624.Contig2_Tc15_2 | 340.8 | 36.2 | Unigene11651_Tc15_2 | 48.0 | 175.8 |
| CL5885.Contig1_Tc15_2 | 132.0 | 48.9 | Unigene11683_Tc15_2 | 24.8 | 0.0 |
| CL6671.Contig1_Tc15_2 | 2.0 | 24.5 | Unigene11935_Tc15_2 | 20.7 | 0.0 |
| CL6956.Contig1_Tc15_2 | 196.2 | 1501.7 | Unigene12071_Tc15_2 | 30.1 | 5.4 |
| Unigene683_Tc15_2 | 41.5 | 8.6 | Unigene12860_Tc15_2 | 45.6 | 15.7 |
| Unigene2822_Tc15_2 | 36.3 | 6.8 | Unigene12905_Tc15_2 | 27.4 | 307.9 |
| Unigene3256_Tc15_2 | 34.5 | 3.2 | Unigene13539_Tc15_2 | 42.2 | 15.4 |
| Unigene3561_Tc15_2 | 127.5 | 59.3 | Unigene15092_Tc15_2 | 10.5 | 426.8 |
| Unigene3795_Tc15_2 | 212.2 | 41.0 | Unigene15605_Tc15_2 | 123.8 | 31.8 |
| Unigene4156_Tc15_2 | 110.8 | 776.2 | Unigene16387_Tc15_2 | 5331.4 | 14187.2 |
| Unigene4204_Tc15_2 | 991.4 | 237.5 | Unigene17187_Tc15_2 | 626.7 | 1639.0 |
| Unigene4298_Tc15_2 | 318.7 | 4804.0 | Unigene17565_Tc15_2 | 92.0 | 234.8 |
| Unigene4450_Tc15_2 | 758.8 | 155.2 | Unigene17799_Tc15_2 | 25.6 | 168.2 |
| Unigene4469_Tc15_2 | 5.3 | 59.7 | Unigene18229_Tc15_2 | 62.9 | 143.9 |
| Unigene4609_Tc15_2 | 8.5 | 85.4 | Unigene18358_Tc15_2 | 168.2 | 39.7 |
| Unigene4712_Tc15_2 | 141.6 | 68.7 | Unigene21130_Tc15_2 | 721.8 | 1525.6 |
| Unigene5013_Tc15_2 | 111.6 | 353.3 | Unigene22186_Tc15_2 | 199.4 | 42.0 |
| Unigene6906_Tc15_2 | 16.3 | 1.9 | Unigene22680_Tc15_2 | 181.0 | 60.8 |
| Unigene7571_Tc15_2 | 24.9 | 3.1 | Unigene22930_Tc15_2 | 29.5 | 13.2 |
| Unigene8006_Tc15_2 | 145.9 | 554.9 | Unigene23118_Tc15_2 | 208.5 | 87.1 |
| Unigene8115_Tc15_2 | 621.9 | 156.8 | Unigene23402_Tc15_2 | 38.5 | 8.6 |
| Unigene8591_Tc15_2 | 601.1 | 1257.7 | Unigene23624_Tc15_2 | 66.1 | 22.1 |
